# Supplementary material for: Hepatitis C continuum of care: Experience of integrative hepatitis C treatment within a human immunodeficiency virus clinic in Indonesia
Source: PLoS One. 2021 Aug 12;16(8):e0256164. doi: 10.1371/journal.pone.0256164 (PMC8360535; doi:10.1371/journal.pone.0256164)
Supplement: Attachment — Submitted filename: Response to Reviewers.docx [file pone.0256164.s001.docx]

July 22^nd^, 2021

Dear Plos One Editor,

Thank you for the opportunity to revise our manuscript entitled “Hepatitis C Continuum of Care: Experience of Integrative Hepatitis C Treatment within a Human Immunodeficiency Virus Clinic in Indonesia”. The constructive comments from reviewers really helped us to improve content of this manuscript.

We have no conflicting interest in writing this manuscript and no financial disclosure need to be done since this study was not received any grant.

We hope that you may find the article in this revised state as satisfactory. We look forward to further feedback or editorial guidance as necessary.

Kind regards,

Evy Yunihastuti

Reviewer #1:

1. The Title could be modified to read “Hepatitis C Continuum of Care: Experience of Integrative Hepatitis C Treatment within a Human Immunodeficiency Virus Clinic in Indonesia”, this reads better with same number of words.

*We really appreciate your valuable suggestion. We have changed the title according to your suggestion.*

2. The sentence on page 15 line 53 “This study was carried out in a specific clinical care of HCV treatment within an integrated HIV clinic in a university hospital in Jakarta” is not clear enough. It is difficult to tell what the authors are attempting to convey with that sentence. This sentence will need to be revise to improve clarity.

*Thank you for pointing this out. We changed this sentence to improve clarity. This study was carried out in HIV integrative clinic of a university hospital in Jakarta. (page 5 line 54)*

3. I suggest the authors rather than use predictors of treatment failure consider the term correlates of treatment failure. The term predictors may suggest some causality, this study is however only able to determine associations and not causality. Correlate is thus a more accurate term for the identified variables in such an analysis.

*Thank you very much for the insightful critique. We understand that this study was unable to determine causality. Therefore, we have changed the term predictor to factor correlated with treatment failure in the whole manuscript.*

4. On page 8 and 9 line 127 – 129, I suggest the authors change that sentence suggesting interaction with all available, HCV drugs. This statement is based on mechanism of action some of these interactions have not been formally studied. A more accurate phrase will be “………. most available DAA drugs”.

*Thank you for your detailed observation. We have changed the sentence according to your suggestion. Rifampicin is known to interact with most available DAA drugs. (page 8 line 127-129)*

5. In the manuscript the authors quote a SVR12 rate of 27.8% for their analysis and a 9.5% rate for Indonesia. This numbers appear to be the successful treatment rate from the cascade. Using the term SVR12 which must only be used to describe successful treatment in those who received HCV antivirals is incorrect. The effective SVR12 rate is 84.7%. This is repeated in many parts of the paper the authors need to make sure they correct that error.

*Thank you for your bringing this point. Studies evaluating HCV continuum of care calculated SVR12 rate in various ways: Boerekamps, et al calculated SVR12 based on patients retained in care; Rizk, et al calculated based on all HCV patients; Saris, et al and Boeke, et al used number of patients with available treatment outcome as denominator; Adekunle, et al used number of patients initiate HCV treatment as denominator of SVR12 rate. We used SVR12 definition the same way as Rizk, et al to define SVR12 rate for the cascade, which was 27.8% (178 of 640 all HIV/HCV co-infected patients). We manually calculated the SVR12 rate for Indonesia in the same way (9.5%: 1635 of 17200 all HCV-infected patients).*

*When evaluating predictor or SOF-DCV treatment failure, the denominator was all patients using SOF-DCV regimen who had treatment outcome data (176 of 184 patients; 95.7%). Two other patients with available treatment outcome were using SOF-SMV and EBR-GZR regimen, not SOF-DCV.*

*We have changed the method section (line 79-96) and added the denominator for each calculation of SVR12 as your suggestion (line. 164-166 and 198)*

6. The reported SVR12 of 95.7% is higher than what I calculated of 84.7% based on all patients who initiated treatment. That is the method used in most analysis, I would suggest the authors clearly define SVR in their analysis since it appears to be different from standard practice. Also, since this calculation is different from other studies comparing with phase III studies and other reports is really not a fair and accurate statement. The authors could do multiple analysis for SVR, but it needs to be clear what the denominator is for each calculation.

*Thank you for the insightful suggestion. We have added the denominator for each calculation of SVR12 according to your suggestion (line 164-166 and 198). We did two different analysis for SVR12. First, SVR12 rate as part of HCV continuum of care cascade, which used HCV antibody positivity as denominator. Second, when evaluating predictor or SOF-DCV treatment failure, the denominator was all patients using SOF-DCV regimen who had treatment outcome data (176 of 184 patients; 95.7%). This was considered the effective SVR12 of SOF-DCV that can be compared with the results of phase III studies and other reports.*

7. The manuscript authors will require to work closely with an English language editor to ensure the message provided in this manuscript is accurate to avoid errors in messaging due to difficulties with English.

*Thank you for your suggestion. We have worked closely with English editor and native speaker for this correction.*

Reviewer #2:

Authors accomplished all suggestions made by the reviewers. There are still some limitations, but these cannot be corrected (as suggested in the Discussion). Thanks for your effort and your time.

*We appreciate the reviewer for the positive response and all suggestions for our manuscript.*
